# Adaptive Gelatin Microspheres Enhanced Stem Cell Delivery and Integration With Diabetic Wounds to Activate Skin Tissue Regeneration

**DOI:** 10.3389/fbioe.2022.813805

**Published:** 2022-04-01

**Authors:** Ming Shi, Yunfen Gao, Lim Lee, Ting Song, Jianhua Zhou, Ling Yan, Yan Li

**Affiliations:** ^1^ Guangdong Provincial Key Laboratory of Sensor Technology and Biomedical Instrument, School of Biomedical Engineering, Shenzhen Campus of Sun Yat-sen University, Shenzhen, China; ^2^ Guangdong Provincial Engineering and Technology Center of Advanced and Portable Medical Devices, Sun Yat-sen University, Guangzhou, China; ^3^ Department of Scientific Research Center, The Seventh Affiliated Hospital, Sun Yat-Sen University, Shenzhen, China; ^4^ Department of Plastic and Cosmetic Surgery, The Third Affiliated Hospital, Sun Yat-sen University, Guangzhou, China

**Keywords:** adipose-derived stem cells, gelatin microspheres, diabetic wound healing, tissue regeneration, exosome

## Abstract

The delayed and complicated diabetic wound healing raises clinical and social concerns. The application of stem cells along with hydrogels is an attractive therapeutic approach. However, low cell retention and integration hindered the performance. Herein, gelatin microspheres were fabricated for local delivery of adipose-derived stem cells (from rats, rADSCs), and the effect of rADSCs with microspheres on diabetic wound healing was examined. Uniform, well-dispersed microspheres were fabricated using the microfluidic technique. Due to geometry differences, the proteinase degradation rate for microspheres was four times that of the bulk hydrogel. The obtained gelatin microspheres supported cell's adhesion and proliferation and provided a suitable microenvironment for rADSC survival. For *in vivo* animal tests, rADSCs were labeled with CM-Dil for tracking purposes. Microspheres were well embedded in the regenerated tissue and demonstrated good biocompatibility and an adaptive biodegradation rate. Histological examination revealed rADSC-loaded gelatin microspheres that significantly accelerated wound healing *via* promoting M2 macrophage polarization, collagen deposition, angiogenesis associated with peripheral nerve recovery, and hair follicle formation. Notably, the relative fluorescence intensity around the hair follicle was 17-fold higher than that of the blank group, indicating rADSC participated in the healing process *via* exosomes. Taken together, the rADSC-laden gelatin microspheres provided a promising strategy for local stem cell delivery to improve diabetic wound healing.

## Introduction

Diabetes has become a global epidemic (Wimmer et al., 2019). The high and growing incidence presented considerable challenges to health care systems (Matoori et al., 2021). A major associated complication was poor or delayed wound healing, which may lead to diabetic foot ulcers (Armstrong et al., 2017). The pathogenesis of diabetic wounds was complicated and multifaceted, including neuropathy and microvascular abnormalities (Falanga, 2005). Optimum cutaneous wound healing required a well-orchestrated integration of complex biological and molecular events, including cell migration and proliferation, angiogenesis, extracellular matrix (ECM) deposition, and remodeling. However, this orderly sequence of cellular and molecular events was disrupted in diabetic wounds.

Current clinical treatments for diabetic wounds involved offloading, debridement, eradicating infected tissue, and maintaining moisture microenvironment (Kavitha et al., 2014). These strategies are mainly aimed at a fast wound closure rather than addressing the underlying pathophysiology and usually result in wound recurrence, leading to treatment failure and maybe amputation (Cavanagh et al., 2005; Falanga, 2005). Numerous approaches such as applying growth factors, anti-inflammatory drugs, matrix metalloproteinase (MMP) inhibitors, scaffolds, and injection of stem cells on diabetic wounds have been evaluated. However, limited performance was reported (Patel et al., 2019). Due to the multifactorial origins of wound etiology, there were no simple methods that aimed at the persistence of wounds in diabetic patients.

Stem cell-based therapy was an attractive approach for the treatment of chronic nonhealing wounds (Dehkordi et al., 2019). There was a growing interest in the potential of adipose-derived stem cells (ADSCs) for diabetic wound healing applications. ADSCs have been shown to promote revascularization and reepithelialization through secretion of proangiogenic factors, such as epidermal growth factor and vascular endothelial growth factor (Kuo et al., 2016), and also have the potential to activate local stem cell niches, reduce oxidative stress, and modulate immune responses (Takemitsu et al., 2012). Combined with the fact that they were easy to isolate, relatively abundant in fatty tissue, and harvested in large numbers with minimal donor site morbidity (Kokai et al., 2014), ADSCs demonstrated a great potential to be used for diabetic wounds. A local injection that directly delivered ADSCs at injury sites causes minimal invasion and thus has gained popularity for clinical applications (Li et al., 2012). However, harsh diabetic wound microenvironment factors such as hypoxia, ischemia, and the persistence inflammation result in upregulation of MMPs (Nguyen et al., 2018), leading to low cell retention and engraftment efficiency, which significantly compromised the clinical performance of stem cell therapies.

To address this hurdle, one potential strategy was to suspend cells in a hydrogel matrix that provided a suitable microenvironment. Because of its superior biocompatibility and controllable physical and functional properties, hydrogel has gained extensive attention for medical wound dressing (Yang et al., 2021). Natural ECM protein such as collagen or its denatured form gelatin retained cell adhesion motifs, and MMP-mediated degradability became popular (Santoro et al., 2014). Dong et al. improved the survival ratio of ADSCs in diabetic wounds using a gelatin-based hydrogel delivery system (Dong et al., 2017). Another research that encapsulated ADSCs in gelatin bulk hydrogel displayed a promoting effect on neovascularization and wound closure (Dong et al., 2018). However, when applied on the wound bed, the limited interface between the bulk hydrogel and the wound resulted in poor tissue infiltration and thus a low survival of stem cells. Therefore, the establishment of alternative hydrogel in other geometries as stem cell delivery vehicles was highly demanded.

Recent advances in tissue engineering have demonstrated the versatility and efficacy of hydrogel microparticles as drug and cell delivery depots (Newsom et al., 2019). Compared with the bulk hydrogel, such geometry endowed microspheres with a large surface area that facilitated nutrient and waste diffusion and thereby maintained the viability of encapsulated cells (Chen et al., 2006). Microparticles also provided mechanical support to the regenerated tissue. Such function was inherently affected by the degradation (Qian et al., 2018). Hence, adaptive material was needed, of which the degradation rate should match well with the tissue regeneration. In addition, our previous research demonstrated that, after being applied on the wound surface, hydrogel microparticles functioned as scaffolds and were gradually embedded in the regenerated skin tissue (Shi et al., 2019a). This finding suggested that hydrogel microspheres could be a suitable carrier for local delivery of ADSCs to improve diabetic wound healing. Although various bulk hydrogels have been studied, microspheres such as gelatin hydrogel microspheres entrapping ADSCs have rarely been applied on wounds. Furthermore, the mechanism of gelatin microspheres along with the encapsulation of ADSCs on diabetic wound healing was still not clear.

Hence, the performance of stem cell therapy together with gelatin hydrogel microspheres on diabetic wound healing was investigated. Gelatin was selected because it had MMP-mediated degradability (Santoro et al., 2014), whereas diabetic wounds were rich in MMPs (Nguyen et al., 2018). Gelatin microspheres were fabricated to encapsulate ADSCs from SD rats (rADSCs) using the microfluidic technique. The properties and the feasibility of gelatin microspheres as a stem cell delivery platform were systematically characterized, including cross-linking degree, proteinase degradation, adhesion and proliferation behaviors of fibroblast cells, and viability and morphology of encapsulated rADSCs. Finally, *in vivo* performances were evaluated in the full-thickness skin diabetic wound model; CM-Dil-labeled rADSCs were encapsulated in microspheres to investigate whether the transplanted rADSCs were involved in the healing process.

## Materials and Methods

### Materials

Gelatin (type A) was obtained from Sigma-Aldrich (St. Louis, MO, USA). Genipin was purchased from Wako (Japan). Dulbecco's modified eagle medium—basic (DMEM), penicillin/streptomycin antibiotics, fetal bovine serum (FBS), collagenase type I, and trypsin–ethylenediaminetetraacetic acid were supplied by Gibco (Carlsbad, CA, USA). Endothelial cell medium was purchased from ScienCell Research Laboratories (Carlsbad). Peanut oil was from Arawana (Guangzhou, China). Ninhydrin, SnCl·2H_2_O, ethylene glycol monomethyl ether, and streptozotocin (STZ) were from Macklin (Shanghai, China). Actin cytoskeleton, focal adhesion staining kit (FAK-100), and pentobarbital sodium were from Merck (Billerica, MA, USA). Cell Counting Kit-8 (CCK-8) was purchased from Dojindo (Shanghai, China). Cell Tracker TM CM-DiI (C7000) was from Molecular Probes (Eugene, USA). β3-tubulin (D71G9) rabbit mAb and Neurofilament-L (DA2) mouse mAb were purchased from Cell Signaling Technology (Danvers, MA, USA). All other chemicals and reagents were of analytical grade. All reagents were used as received.

### Preparation of Uniform Gelatin Microspheres

Monodisperse gelatin microspheres were fabricated using a microfluidic device consisting of two syringe needles and a polytetrafluoroethylene tube (inner/outer diameters were 0.3 and 0.6 mm, respectively). Briefly, a 26 G needle was first connected with the polytetrafluoroethylene tube, and then, a 31 G needle was inserted into the tube at an appropriate position, keeping the needle concentric within the tube. All connections were sealed with acrylic adhesive glue. The internal phase was gelatin/phosphate-buffered saline (PBS) solution (10% w/v, 37°C), and the flow rate was set as 5 μl/min. Peanut oil was the continuous phase, at a flow rate of 100 μl/min. Gelatin microspheres were collected in 10°C peanut oil for 10 min, and then, these batch microspheres were further incubated in 4°C ice bath for another 10 min for complete gelation. Genipin/PBS solution (0.5% w/v, 1 ml) was added into the microsphere/peanut oil suspension (containing 50-mg microspheres) at room temperature (∼23°C) for cross-linking. After centrifugation to remove the supernatant, microspheres were rinsed three times with DMEM (containing 5% FBS). The collected microspheres were stored in PBS for subsequent characterizations.

To obtain sterile microspheres, gelatin/PBS solution, peanut oil, genipin solution, and PBS were sterilized by filtration, and all fabrication procedures were performed in a laminar flow hood.

### Characterizations of Gelatin Microspheres

The cross-linking degree of gelatin microspheres was quantified using a ninhydrin assay, which was defined as the percentage of amino groups reacted with the cross-linking agent (Liang et al., 2003). One milliliter of ninhydrin solution was added into each sample containing 50-mg gelatin microspheres, which were cross-linked with genipin for 0, 1, 2, or 4 h (*n* = 3). After incubated at 100°C for 20 min, isopropanol (50% w/v, 5 ml) was added, and 1-ml supernatant was taken out to measure the absorbance at 570 nm. For the following tests, microspheres cross-linked for 1 h were used.

*In vitro* proteinase degradation of microspheres was monitored following similar procedures reported elsewhere (Turner et al., 2017). In brief, microspheres (100 mg/ml) were suspended in collagenase type I solution (0.75 mg/ml) at 37°C. Because the reaction between genipin and amino acids formed blue molecules, which showed the maximum absorbance at 590 nm and also released into the solution due to proteinase degradation of gelatin, absorbance at 590 nm of the supernatant was measured every 90 s.

The morphology of hydrogel microspheres was observed under a light microscope, and the average particle size was calculated using ImageJ. After freeze-drying, surface morphology was observed using a scanning electron microscope (Quanta 400F, FEI, Netherlands).

Adhesion and proliferation behaviors of fibroblast cells (NIH-3T3) on sterile gelatin microspheres were investigated. Microspheres were placed in a non-treated 48-well plate (50 mg/well), whereas a treated 48-well plate was used as the control group (TCP). NIH-3T3 cells were seeded at a density of 5 × 10^4^ cells/well. On days 1 and 3, cell viability was measured using a CCK-8 assay (*n* = 3). Cytoskeleton staining was carried out on day 2, following the manufacturer's instructions. Images were taken using a confocal laser scanning microscope (CLSM, FV3000, Olympus, Japan).

### Preparation and Characterizations of Adipose-Derived Stem Cells From Rats-Loaded Gelatin Microspheres

rADSCs were isolated from rats following our previous procedures (Shi et al., 2019b). Cells at passages 3–4 were mixed with gelatin/PBS solution. In the suspension, gelatin concentration was 10%, and cell density was 5 × 10^6^ cells/ml. A total of 100 μl of cell suspension was loaded in a syringe as internal phase, and other parameters were followed as described in *Preparation of Uniform Gelatin Microspheres*. After rinsing, rADSC-loaded gelatin microspheres (rADSC/MS), which contained 5 × 10^5^ cells per sample, were cultured in DMEM/FBS medium. On days 1 and 7, live/dead assay and cytoskeleton staining were performed and observed under CLSM.

For *in vitro* coculture and *in vivo* tests, rADSCs were labeled with CM-Dil before being entrapped in gelatin microspheres. In brief, 1 mg/ml CM-Dil stock solution was diluted with PBS at 1:500. rADSCs were rinsed with PBS. After adding CM-Dil solution, cells were incubated for 30 min at room temperature and then kept in a refrigerator at 4°C for 15 min to enhance CM-Dil labeling the plasma membrane. After the staining process, the cells were rinsed with PBS.

CM-Dil-labeled rADSC/MS were cocultured with human umbilical vein endothelial cells (HUVECs, a gift from Quan Daping group in School of Materials Science and Engineering, Sun Yat-sen University) to investigate whether exosomes from rADSCs were uptaken by HUVECs. HUVECs were seeded on Petri dishes for CLSM at a density of 5,000 cells/cm^2^ and cultured in an endothelial cell medium. CM-Dil-labeled rADSC/MS were placed in the transwell. After cocultured in the endothelial cell medium for 5 days, HUVECs were fixed, stained with 4′,6-diamidino-2-phenylindole, and observed under CLSM. HUVECs being solo-cultured were also observed.

### Application of Gelatin Microspheres on Full-Thickness Skin Wound of Type I Diabetes Mellitus Rats

The *in vivo* test was approved by the Institutional Animal Care and Use Committee of Sun Yat-sen University. Type I diabetes mellitus (TIDM) was induced in Sprague-Dawley rats (SD rats, 250–300 g, 8 weeks old) according to routine procedures (Kuo et al., 2009). Briefly, after fasting for 1 day, tail vein blood was taken to measure the fasting blood glucose. STZ solution (1% w/v) was injected intraperitoneal at 50 mg/kg. The model was considered successfully induced when the initial blood glucose value was <8.9 mmol/L and the random blood glucose after injection of STZ solution was ≥16.7 mmol/L for three consecutive days.

TIDM rats were randomly divided into three groups (*n* = 5). After anesthetized with 3% sodium pentobarbital solution, three circular full-thickness excisions with a diameter of 8 mm were created on the back of each rat. MS (100 mg) and rADSC/MS (100 mg containing 5 × 10^5^ cells) were applied on wound beds; wound without any treatment was set as the control group. Wounds were then covered with a transparent dressing and further fastened with a self-adhesive bandage for protection. On days 3, 7, and 14, wounds were photographed using a digital camera, and ImageJ was used to measure the wound area. The wound contraction was calculated using Eq. 1:

$$Wound\;contraction\;\left(\%\right)=\left(A_{0}-A_{t}\right)/A_{0}\times 100\%$$ (1)

where A_0_ is the wound area on day 0, and A_t_ is the wound area at the indicated time point.

On days 3, 7, and 14, rats were killed by injection of excessive 3% sodium pentobarbital solution. Optical coherence tomography (OCT) analysis was first used to observe the distribution of gelatin microspheres in the wound bed. The scanning wavelength was 1,310 nm.

### Histological and Immunohistochemistry Staining Analysis

The full-thickness skin around the wound (1.5 × 1.5 cm) was removed, fixed in paraformaldehyde solution, paraffin-embedded, and sectioned for hematoxylin/eosin staining, and Masson's Trichrome staining and CD31 immunohistochemical staining were performed as previous (Shi et al., 2019a). Sectioned tissue without any staining was observed under CLSM to investigate whether CM-Dil-labeled cells were present.

### Immunofluorescence Analysis

For neuronal marker staining, sections were first repaired using ethylenediaminetetraacetic acid antigen retrieval solution (pH 9.0) for 5 min at 100°C and blocked with 3% BSA solution for 30 min. After rinsing, sections were probed overnight at 4°C with the following primary antibodies: β3-tubulin (1:200, Cell Signaling Technology) or Neurofilament-L (1:100, Cell Signaling Technology). Sections were then washed and incubated with Alexa Fluor 488 conjugated goat anti-rabbit or rabbit anti-mouse secondary antibodies (1:500, Invitrogen) for 1 h at 37°C, correspondingly.

To investigate the inflammatory response, immunofluorescence analysis of CD68 (1:1,000, Invitrogen) and CD206 (1:200, Proteintech) was performed similarly as earlier. Sections were then incubated with Alexa Fluor 647 conjugated goat anti-rabbit or Alexa Fluor 568 conjugated rabbit anti-mouse secondary antibodies (1:500, Invitrogen) for 1 h at 37°C, respectively.

To evaluate exosomes, CD63 primary antibody (1:200, Affinity) was selected and incubated with sections at 4°C overnight, then treated with Alexa Fluor 647 conjugated goat anti-rabbit secondary antibody.

Nuclei were counterstained with 4′,6-diamidino-2-phenylindole for 10 min. Sections were observed using a CLSM.

### Statistical Analysis

All data were shown as mean ± standard deviation, and the difference between groups was analyzed with Student's paired T-test. *p* < 0.05 indicated that difference was statistically significant.

## Results

### Characterizations of Gelatin Microspheres

**FIGURE 1:**
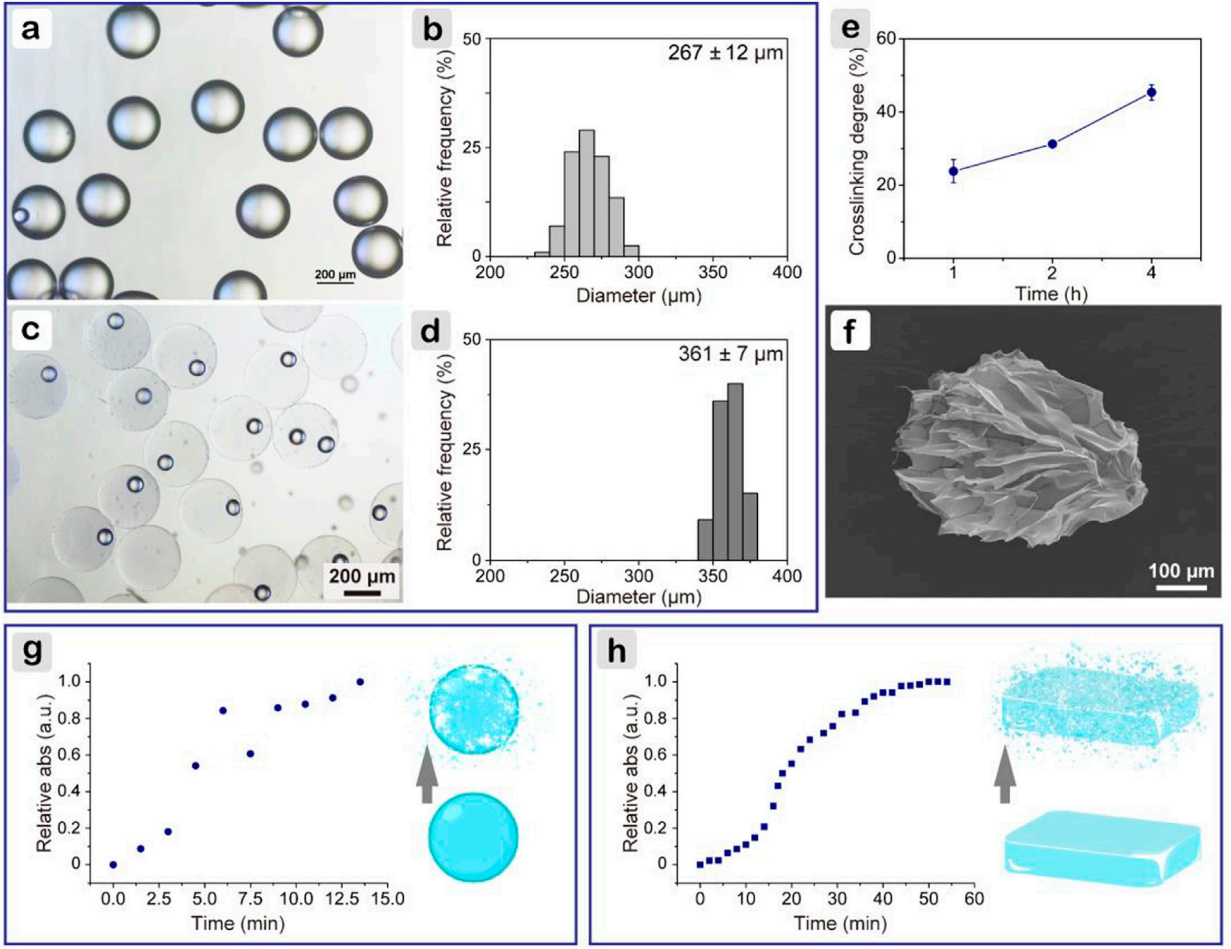
Characterizations of gelatin microspheres. **(A)** Light microscope image of microspheres in precooled peanut oil, and **(B)** corresponding particle size distribution; **(C)** after cross-linking for 1 h, and **(D)** corresponding particle size distribution; **(E)** cross-linking degree of gelatin microspheres; **(F)** scanning electron microscope image of gelatin microsphere after lyophilization; change of relative absorbance at 590 nm for microspheres **(G)** and bulk hydrogel **(H)** with proteinase degradation time.

Gelatin microspheres were prepared using a microfluidic method. Precooled peanut oil (∼10°C) was used as the collecting phase to rapidly gelatinize microdroplets. As shown in Figures 1A,B, uniform microspheres with a narrow size distribution were obtained, for which the average diameter was 267 ± 12 μm. Then, genipin/PBS solution was added to cross-link microspheres. This introduced a nucleophilic agent to initiate the cross-linking reaction of genipin with itself, thereby forming an oligomeric cross-linking agent that produces a high degree of intermolecular binding (Liang et al., 2004). The reaction was carried out at 23°C to ensure that the precooled microspheres were not dissolved and cross-linked at an appropriate rate and formed a stable structure; when compared with 10 or 4°C, cross-linking temperature of 23°C had minimum influences on cell viability. As shown in Figures 1C,D, after reaction for 1 h, gelatin microspheres were still well-dispersed and stable at 37°C. The average diameter was 361 ± 7 μm, slightly bigger than that in the oil phase. Based on the ninhydrin assay (Figure 1E), it was found that the cross-linking degree positively correlated with the cross-linking time. Because cells showed a more spread morphology within soft hydrogel matrix (Sung et al., 2018), 1 h was selected. After lyophilization, scanning electron microscope image (Figure 1F) showed that the particle was no longer spherical, whereas the size was slightly increased. This may be due to the freeze-drying process. The lyophilized microspheres had a mesh-liked surface, indicating that microspheres were porous.

Gelatin microspheres were developed to be applied on diabetic wounds rich in MMPs, such as MMP2 and MMP9 (Gooyit et al., 2014). As MMPs and collagenase shared common target sites to cleave amide linkages within the repeating peptide motif QPQGLAK in gelatin (Fonseca et al., 2014), collagenase (type I) was used to compare the degradation rates of gelatin hydrogel in different shapes. The prepared microspheres were stable in PBS longer than 7 days. When collagenase was introduced, the degradation was much faster. As shown in Figure 1G, within 10 min, microspheres were completely degraded. The rate was higher than that reported in the literature, where microspheres were degraded within 14 min (Turner et al., 2017). The difference was probably because of the slightly lower cross-linking degree for the microspheres here. As for the bulk hydrogels, which were fabricated following the same parameters for microspheres, fourfold longer time (*i.e.*, 44 min) was needed (Figure 1H). Microspheres had a much higher specific surface area than bulk hydrogels, which facilitated enzyme infiltration and further contributed to a faster degradation rate.

### *In Vitro* Cell Tests for Gelatin Microspheres as Adipose-Derived Stem Cells From Rats Delivery Platform

**FIGURE 2:**
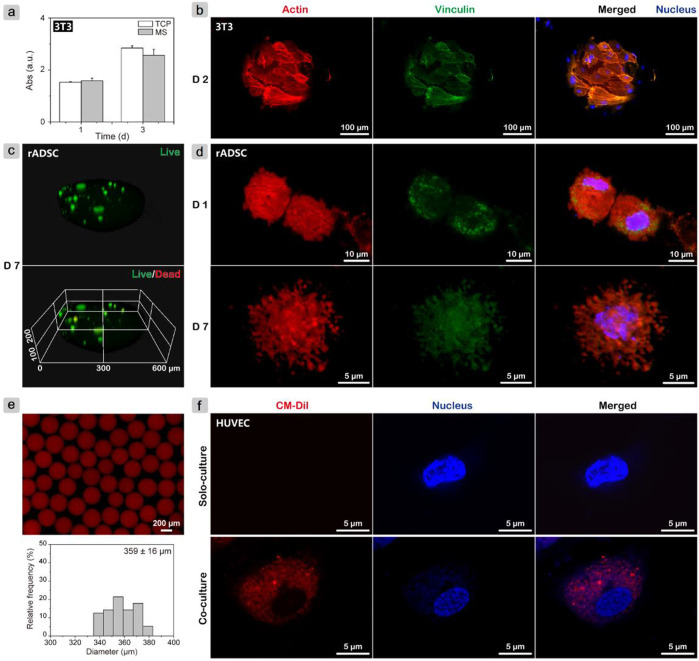
*In vitro* cell test results. **(A)** Monitoring proliferation of NIH-3T3 cells on gelatin microspheres using CCK-8 assay; **(B)** CLSM images of NIH-3T3 cells after growing on microspheres for 2 days and cytoskeleton staining to evaluate adhesion behavior. **(C)** Live/dead assay and **(D)** cytoskeleton staining of rADSCs inside gelatin microspheres after culturing for 7 days to investigate cell viability and spreading morphology, respectively. **(E)** Fluorescence image of CM-Dil-labeled rADSC/MS and corresponding particle size distribution. **(F)** CLSM images of HUVECs after cocultured with CM-Dil-labeled rADSC/MS and solo-cultured for 5 days.

The proliferation rate of fibroblasts (NIH-3T3) was measured using the CCK-8 assay (Figure 2A), where the MS group showed close values with the TCP group on days 1 and 3. Cell adhesion behaviors were further investigated. After growing for 2 days, all cells spread well on the microsphere surface (Figure 2B). The well-organized actin filament structure and presence of vinculin were observed. On gelatin microspheres that were fabricated using the double emulsion method and also cross-linked with genipin, osteoblasts on large microspheres (200–300 μm) had a larger spread out morphology and a faster proliferation rate than on small microspheres (75–160 μm) during 7 days of culture (Lau et al., 2010). Thus, also considering the particle size effect on cell proliferation, it was reasonable that gelatin microspheres here supported cell adhesion and proliferation.

To determine the suitability of gelatin microspheres as delivery vehicles for stem cells, live/dead assay and cytoskeleton staining for the encapsulated rADSCs were conducted. After culturing for 7 days and live/dead staining, microspheres were scanned with CLSM for 100 layers, and a three-dimensional (3D) image was then reconstructed. As shown in Figure 2C, all rADSCs were green, whereas nearly no red cells were detected, demonstrating that most cells were still viable. On the first day, the cells were spherical; actin without filament structure was detected, whereas vinculin structures were present. After 7 days of culture, rADSCs spread out, and a large number of filopodia structures formed (Figure 2D), which may be the result of the gradual interactions between cells and gelatin matrix (Baeckkyoung et al., 2018). The changes of cell spreading morphologies with culture time were similar to the reported results that within soft gelatin matrix, development of filopodia and actin filament in stem cells was easier than that within the rigid matrix (Zhao et al., 2016; Sung et al., 2018).

The fabricated gelatin microspheres did not only facilitate the adhesion and proliferation of fibroblast cells but also maintained the cell viability of the encapsulated rADSCs. For MSCs, the therapeutic potential to accelerate cutaneous wound healing was found to be related to exosomes (Shabbir et al., 2015). Red fluorescent lipophilic dye (Dil)-labeled exosomes from Dil-labeled synovium MSCs were found to be in the perinuclear region of human dermal microvascular endothelial cells (Tao et al., 2017). To investigate whether exosomes from rADSCs in gelatin microspheres were internalized by other types of cells, rADSC/MS with CM-Dil-labeled rADSCs were fabricated. As shown in Figure 2E, the gelatin matrix demonstrated autofluorescence property at ∼600 nm because of cross-linking (Solorio et al., 2010). When compared with blank microspheres, the one entrapping cell showed a broader size distribution. Coculture was carried out in HUVEC medium (ECM medium), in which rADSCs also proliferated well (Supplementary Figure S1). After 5 days, red fluorescent exosomes were observed inside HUVECs (Figure 2F). This phenomenon confirmed that exosomes were slowly released from rADSCs and eventually internalized by HUVECs seeded on TCP. The release and uptaking of exosomes may improve diabetic wound healing.

### Adipose-Derived Stem Cells From Rats Improving Diabetic Wound Healing

**FIGURE 3:**
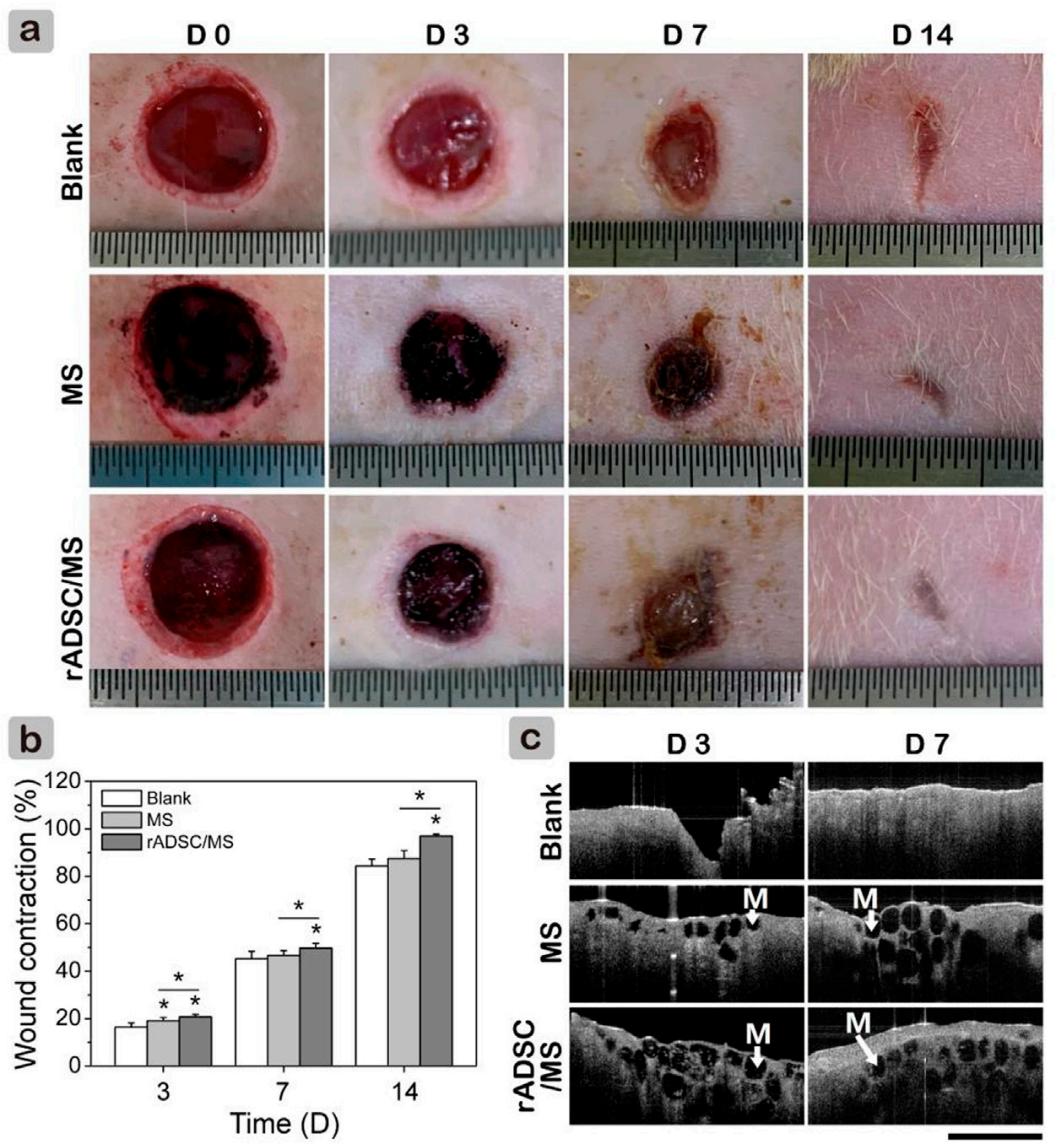
Effects of gelatin microspheres on wound healing in diabetic rats. **(A)** Wound healing process within 14 days was recorded in photographs. Length between adjacent lines on ruler is 0.5 mm. **(B)** Wound contraction was calculated based on these images (data = mean ± SD, *n* = 5, *p < 0.05). **(C)** OCT scanning images for section view of regenerate granulation tissue. Black circles (also “M”) in wound area represent gelatin microspheres. Scale bar is 2 mm.

Full-thickness wounds of TIDM rats were established to evaluate the performance of rADSC/MS on wound healing. The digital images of wounds within 14 days are shown in Figure 3A. Microspheres attached wound beds tightly. When compared with the blank group, both the MS and rADSC/MS groups appeared to have less bleeding; during the following healing process, these groups showed a more rapid wound contraction. On day 14, the rADSC/MS group nearly completely healed with regenerated smooth skin, whereas the blank group only healed ∼80%. At each time point, the contraction value for the rADSC/MS group was significantly higher than that for the MS and blank groups (Figure 3B). To detect microsphere integration, OCT was used to scan the wounds *in situ*. As shown in Figure 3C, the regenerated tissue was around and over microspheres in both the MS and rADSC/MS groups, showing that microspheres were gradually integrated into the regenerated skin tissue from days 3–7.

**FIGURE 4:**
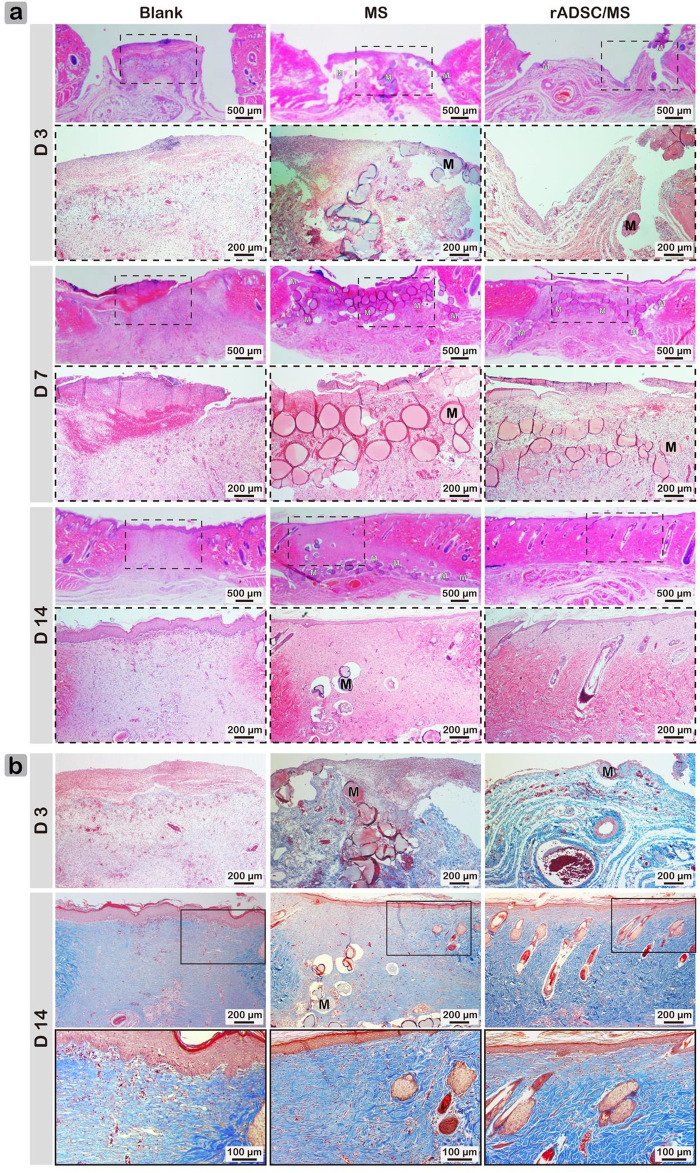
Representative **(A)** hematoxylin/eosin- and **(B)** Masson's Trichrome-stained wound sections for blank, MS, and rADSC/MS groups on days 3, 7, and 14. “M” represents gelatin microspheres. Microspheres functioned as scaffolds to fasten wound healing and enhance collagen deposition; with encapsulation of rADSCs, enhancing effect was more significant. On day 14, arrangement of collagen fibers for rADSC/MS group was similar to unwounded skin.

Macroscopic results demonstrated that rADSC/MS accelerated diabetic wound contraction. To gain further insights, histological analysis was performed. As shown in Figure 4A, on day 3, granulation tissue was observed in all groups, and a few microspheres were detected for both the MS and rADSC/MS groups. The bonding between granulation tissue and microspheres was not strong enough; therefore, the distribution of microspheres on wound sections was probably affected by the embedding and following processes. On day 7, granulation tissues grew around microspheres in both the MS and rADSC/MS groups. A migrating reepithelialization layer from the wound edge to the center gradually formed over the microspheres. This trend was evident for the rADSC/MS group, demonstrating that the wound was in a rapid healing rate. Neutrophils clustered at the microsphere edge, showing a certain inflammatory response. On day 14, both epidermis and dermis layers were reestablished for all groups, whereas the structure of the dermis layer was different. For rADSC/MS, the regenerated dermis was close to the normal tissue; plenty of growing hair follicles and sebaceous glands were present, and no layers of remaining microspheres were detected. As for the MS group, hair follicles and sebaceous glands gradually formed from wound edges over undegraded microspheres. For the blank group, there was no hair follicle or sebaceous gland observed, indicating that the wound healing was much slower. Additionally, in subdermal layers of the MS and rADSC/MS groups, there were much more large blood vessels than the blank group.

Collagen is susceptible to glycosylation, which reduces its sensitivity to enzymes and further affects metabolism, repair, and renewal (Abiko and Selimovic, 2010). Factors such as increased level of MMP9 in diabetic skin impair collagen accumulation leading to impaired wound healing (Li et al., 2013). Therefore, Masson's Trichrome staining was performed to determine the formation and distribution of collagen fibers (Figure 4B). The blank group always showed the least collagen production. Compared with the MS group, collagen deposition in the rADSC/MS group was more significant. On day 14, dense collagen formed, and collagen fibers arranged in a neat manner similar to normal tissues, whereas neither the MS group nor the blank group had mature collagen fibers.

### Mechanism of Adipose-Derived Stem Cells From Rats on Improving Diabetic Wound Healing

**FIGURE 5:**
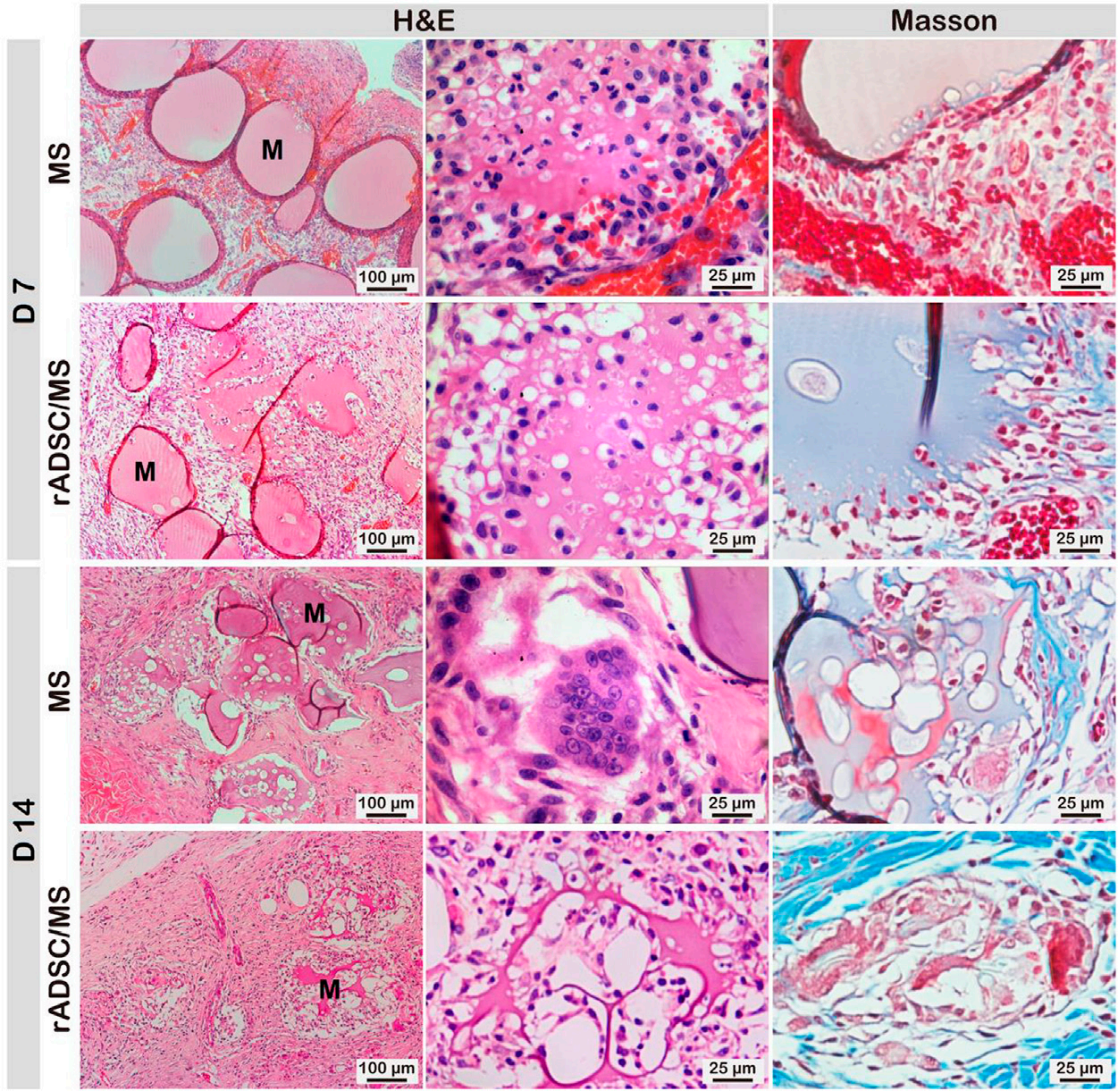
Biocompatibility and biodegradability of gelatin microspheres during wound healing. Degradation rate of microspheres in rADSC/MS group appeared to be faster than that in MS group. “M” represents gelatin microspheres.

During wound healing, gelatin microspheres were embedded in the regenerated skin tissue for both the MS and rADSC/MS groups. Gelatin is hydrolyzed from collagen, with good biocompatibility and biodegradability (Sivadas et al., 2008). As shown in Figure 5, the degradation of gelatin microspheres in the rADSC/MS group was faster. On day 7, nearly intact microspheres with neutrophils gathering on the surface were present in the MS group; for the rADSC/MS group, a lighter inflammation response was detected, and most microspheres became porous. Such a porous structure may facilitate the signaling between rADSCs and the host tissue. On day 14, in the MS group, microspheres became porous, whereas microspheres of the rADSC/MS group were basically degraded. Besides neutrophils, macrophages also took part. Regarding the faster degradation rate for the rADSC/MS group, entrapping rADSCs may result in slightly higher water content in microspheres, as gelatin hydrogel with a higher water content degraded faster when subcutaneously implanted into mice back (Tabata, 1998). The tissue regeneration rate adaptively matched with the microsphere degradation rate, especially for the rADSC/MS group, where the collagen fibers around microsphere debris arranged neatly.

**FIGURE 6:**
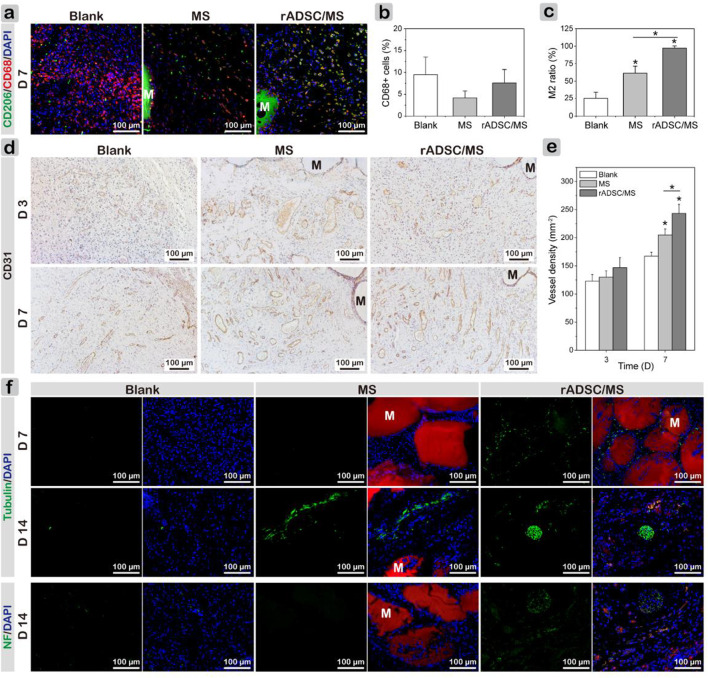
Investigating mechanism of MS and rADSC/MS on promoting diabetic wound healing by evaluating macrophage polarization, angiogenesis, and neurogenesis during wound healing. **(A)** Representative CLSM images for all groups on day 7 showing increased presence of M2 macrophages around microspheres, as evidenced by co-staining of CD68 and CD206. CD68 antibody was conjugated with Alexa Fluor 647 (650/668 nm), and CD206 antibody was conjugated with Alexa Fluor 568 (578/603 nm). **(B)** Percentage of CD68^+^ cells; **(C)** M2 macrophage ratio of CD206 + CD68^+^ cells to CD68^+^ cells. **(D)** Representative CD31 immunohistochemical staining images of wound sections on days 3 and 7 and **(E)** corresponding quantitative results of vessel density. **(F)** Immunofluorescence images of sections revealing distribution of newly regenerated neurons (tubulin) and axon (Neurofilament-L) in all groups on days 7 and 14, respectively. Tubulin and Neurofilament-L antibodies were conjugated with Alexa Fluor 488 (495/518 nm). “M” represents gelatin microspheres. Fluorescence intensity was calculated using ImageJ. Data = mean ± SD, *n* = 3, *p < 0.05 when compared with blank or MS group.

Macrophages were key factors in the inflammatory–proliferation phase transition, which mainly contained two subtypes: classically activated M1 with pro-inflammatory properties and alternatively activated M2 exhibiting anti-inflammatory and pro-wound healing functions (Wynn et al., 2013). On day 7, co-staining results of CD68 and CD206 demonstrated a much higher percentage of M2 macrophages in the regenerated tissues of the rADSC/MS group than the other two groups (Figures 6A,C). This phenomenon indicated that the wound was in the process of healing. As for the blank group, more CD68 ^+^ cells were still present, showing an excessive inflammatory response (Figure 6B). This may illustrate the delayed wound healing. The encapsulation of rADSCs in the rADSC/MS group may regulate the polarization of macrophages to the M2 subtype and thus promote wound healing.

Local ischemia results from microvascular complications, and hyperglycemia inhibits angiogenesis in diabetes, which considerably delays wound healing (Botusan et al., 2008). Thus, the reestablishment of the vascular network is crucial. Immunohistochemical staining of CD31 for tissue sections and quantitative analysis of vessel densities based on CD31-positive capillaries were performed. Figure 6D shows that on both days 3 and 7, a large number of blood vessels regenerated. With healing time, the density of CD31-positive capillaries increased, where on day 7, the value of the MS group was significantly higher than that for the blank group, and the rADSC/MS group had the highest value (Figure 6E). On day 14, vessel density decreased in all groups (Supplementary Figure S2) because vascularization mainly occurred in the early stage of wound healing. These comparisons indicated that gelatin microspheres showed a certain promoting effect on angiogenesis; when rADSCs were entrapped, the promoting effect was stronger (rADSC/MS).

Diabetic peripheral neuropathy is the most common chronic complication (Galuppo et al., 2014). Besides looking at tissue regeneration, it was also important to observe the distribution of peripheral nerves. Thus, immunofluorescent staining of β III-tubulin and Neurofilament-L was conducted (Figure 6F). For the blank group, very few expressions for these two proteins were observed. In the rADSC/MS group, positive expressions of β III-tubulin around gelatin microspheres were observed on day 7. Such protein expression correlates with the early phase of neural differentiation, implying that neurogenesis began. On day 14, a relatively mature bundle of nerve fibers around degraded microspheres in the subdermal layer appeared; some β III-tubulin-positive cells co-fluoresced with red color (Supplementary Figure S3), demonstrating that these cells may be related with rADSCs (CM-Dil labeled). The presence of axon marker (Neurofilament-L) also demonstrated the promoting effects of rADSC/MS on neurogenesis, inducing axon's growth into the wound bed.

**FIGURE 7:**
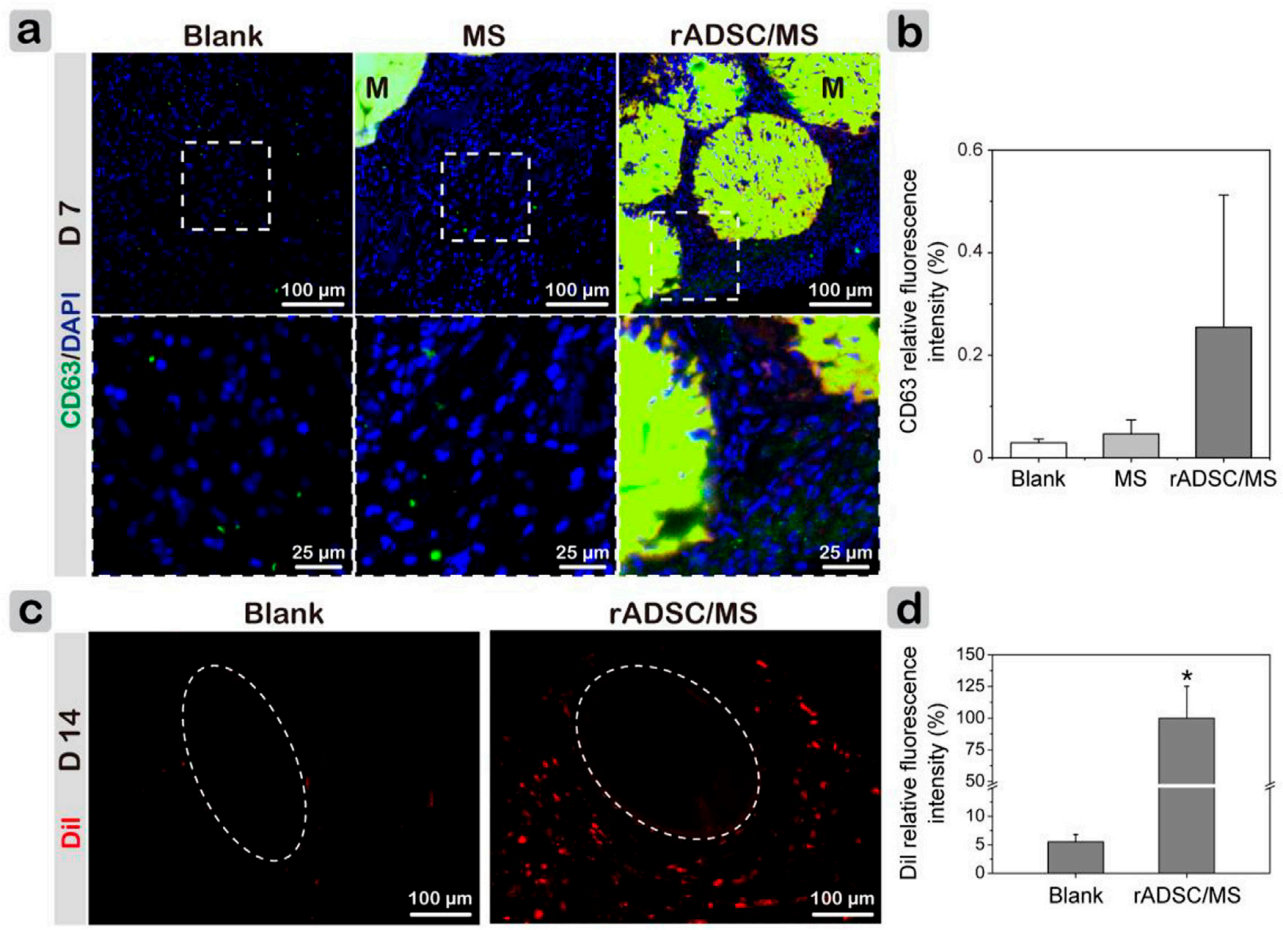
Investigating paracrine performance of encapsulated rADSCs during wound healing. **(A)** CD63 staining results showing presence of exosomes in regenerated tissues on day 7. Local magnified images were also shown. CD68 antibody was conjugated with Alexa Fluor 647 (650/668 nm). “M” represents gelatin microspheres. **(B)** CD63 fluorescence intensity of three groups on day 7. **(C)** CLSM images of wound sections without any staining on day 14. Dotted circles represent hair bulbs in subdermal layer, and rADSCs in gelatin microspheres were labeled with CM-Dil (Dil). **(D)** Corresponding Dil relative fluorescence intensity for blank and rADSC/MS groups. Intensity was calculated using ImageJ. Data = mean ± SD, *n* = 3, *p < 0.05 when compared with blank group.

It was of great interest to investigate whether rADSCs are involved during wound healing. As *in vitro* coculture tests showed that rADSCs released exosomes that were internalized by other cells, immune fluorescence staining of exosome marker CD63 was performed on tissue sections (Nakazaki et al., 2021). On day 3, exosomes were observed in granulation tissues, and the fluorescence intensity of CD63 was slightly higher in the rADSCs/MS group (Supplementary Figure S4). On day 7, exosomes arose between microspheres in rADSCs/MS, and the fluorescent intensity was much higher than in other groups (Figures 7A,B). Here, rADSCs were labeled with CM-Dil. On day 14, as shown in Figure 7C (full-thickness results are shown in Supplementary Figure S5), plenty of red fluorescent cells around hair follicles were observed in the rADSC/MS group, whereas few fluorescent cells were detected in the same area for the blank group. The corresponding fluorescence intensities of the rADSC/MS group were 17-fold higher than that of the blank group (Figure 7D). Furthermore, plenty of fluorescent cells around the undegraded microsphere were also observed in the rADSC/MS group. As for the MS group, remaining autofluorescent gelatin microspheres were present, and no fluorescent cells were around (Supplementary Figure S5). Also, combined with the *in vitro* coculture results that red fluorescent exosomes were observed inside HUVECs, it can be inferred that these entrapped rADSCs remained viable during wound healing, and the paracrine effects may be through exosomes.

## Discussions

**FIGURE 8:**
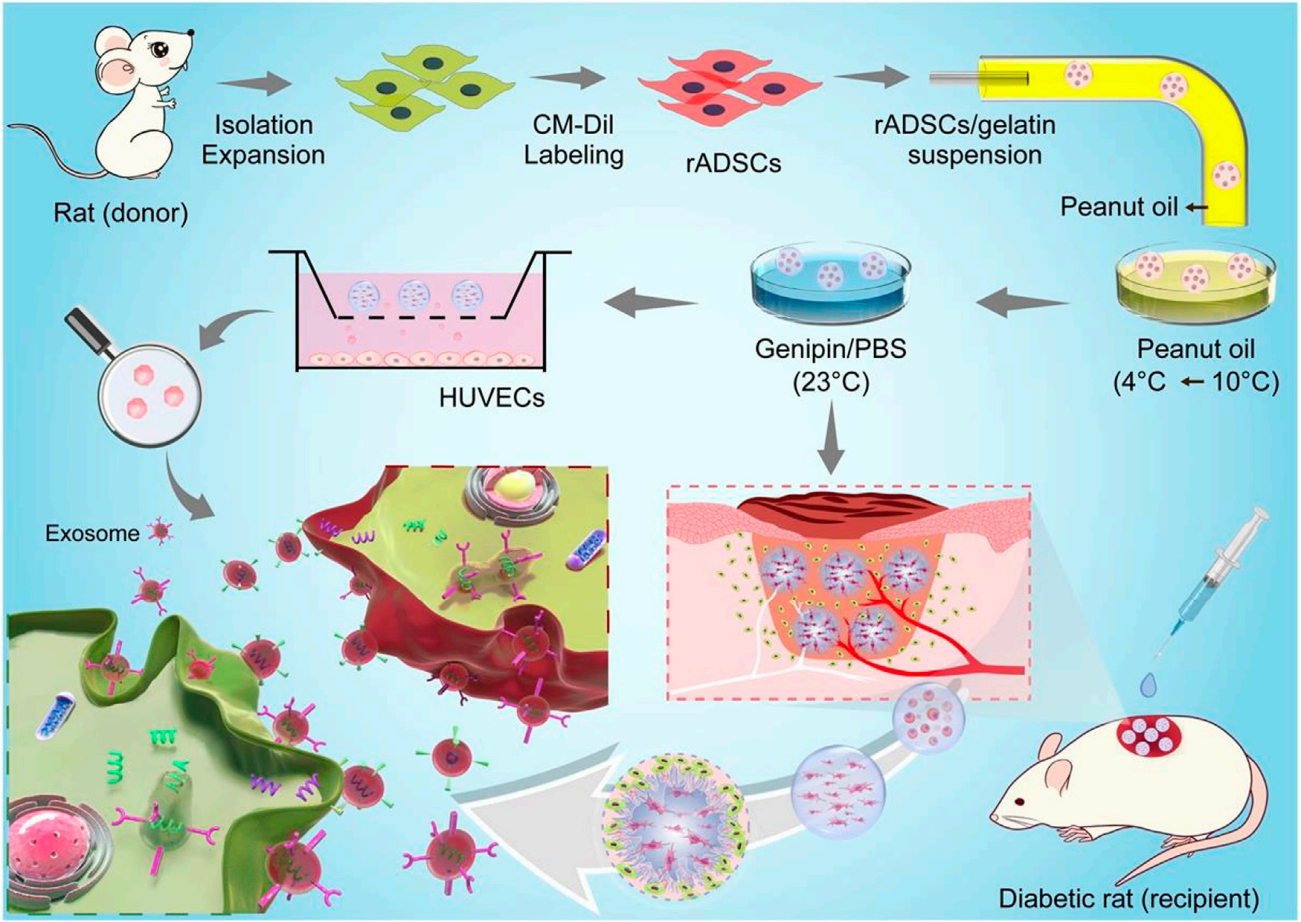
Schemes for preparation of CM-Dil-labeled rADSCs-laden gelatin microspheres and application for diabetic wound healing. rADSCs were isolated and labeled with CM-Dil after expansion and then encapsulated in gelatin microspheres using a microfluidic method. After cross-linking, microspheres were applied in a full-thickness diabetic wound to enhance wound healing, including reepithelialization, angiogenesis (red tube), and nerve regeneration (white tube). Gelatin matrix degraded along with tissue regeneration in wound bed, and entrapped rADSCs influenced surrounding cells *via* paracrine effect. Schematic diagram for paracrine effect was shown on left corner; exosomes were released from CM-Dil-labeled rADSC (red) and uptaken by recipient cell.

In this study, genipin–cross-linked gelatin microspheres were fabricated to deliver stem cells for diabetic wound healing (Figure 8), where the strategy did not only fasten wound closure but also enhanced functional recovery, including revascularization and regeneration of peripheral nerves and hair follicles.

Among various hydrogel materials, gelatin has attracted wide interest as a scaffold material because it retains intrinsic integrin-binding ligands for cell adhesion and can be easily cross-linked with genipin, a plant-derived cross-linker (Bigi et al., 2002). The genipin concentration and cross-linking time were two major factors influencing the viability of encapsulated cells and the elastic stiffness of cross-linked hydrogel. A total of 0.5% w/v genipin in a medium for 60 min maintained ∼87% viability of MSCs, and the value decreased to ∼80% at 1% w/v genipin for only 30 min (Baeckkyoung et al., 2018). Here, using a low concentration of 0.5% w/v cross-linking for 1 h did not only minimize the cytotoxicity of genipin but also resulted in a soft gelatin matrix. Additionally, encapsulation in gelatin microspheres may further protect rADSCs from genipin, as MSCs within 3D gelatin microgels exhibited significantly elevated viability as ∼90% compared with 2D monolayer culture with the viability of ∼55% over 7 days in the pro-inflammatory environment (Baeckkyoung et al., 2018). Here, the high cell viability of rADSCs inside gelatin microspheres ensured the applicability to treat diabetic wounds.

A major challenge in MSC-based therapy for diabetic wound treatment has been associated with poor cell survival and integration (Wu et al., 2010). When injection of ADSC suspension in PEG-gelatin hydrogel into diabetic wounds, a remarkable decrease of ∼40% cell viability as early as 2-h post-transplanting was detected (Dong et al., 2018). The main reasons included mechanical damage during injection, lack of cell-matrix adhesions, and the harmful inflammatory conditions at the injured site (Rustad et al., 2012). Here, encapsulating rADSCs inside gelatin microspheres provided a suitable microenvironment in the early stage of transplantation. After initial integration (Figure 3C), microspheres along with rADSCs well participated in the tissue regeneration (Figure 4). It was reported that GFP-labeled ADSCs migrated and accumulated in the subdermal layer of the wound margin on day 10 after injection and expressed vascular endothelial growth factor in the peri-wound area to activate neoangiogenesis. However, no fluorescent cells were observed around accessory organelles in the cutaneous layer (Kuo et al., 2016). It seemed that the local existence of ADSCs was crucial. Here, when rADSCs/MS were applied on diabetic wounds, gelatin microspheres were well integrated into the regenerated tissue to avail full local utilization of the entrapped rADSCs.

To restore tissue function, scaffold degradation was required to balance with tissue regrowth (Griffin et al., 2021). Here, gelatin microspheres may attract MMPs within diabetic wounds, resulting in the local enrichment of MMPs and microsphere degradation. The speculation was supported by the significant difference that gelatin microspheres were stable longer than 7 days in PBS, whereas *in vivo* microspheres became porous on day 7. As for the rADSC/MS group, microspheres were nearly completely degraded on day 14. This was much faster than the reported result, where the degradation time of gelatin hydrogel containing ADSCs was ∼28 days (Dong et al., 2018). Compared with bulk hydrogel, the degradation of microspheres was faster. This was confirmed by the *in vitro* results that 10 min for microspheres compared with 44 min for bulk hydrogels were needed to degrade completely in collagenase solution. An appropriate degradation rate of scaffolds may guide fibroblasts infiltration, promote granulation tissue formation, and also prevent excessive contraction and fibrosis (Nyame et al., 2015), thus resulting in smooth skin for the rADSC/MS group.

Gelatin microspheres were well integrated into wounds and gradually degraded during tissue regrowth. ADSCs have been shown to secrete massive growth factors to improve fibroblast proliferation (Kim et al., 2011). Such paracrine effects were affected by the distance between MSCs and surrounded cells. The value was reported to be less than 200 μm to preferentially promote tube formation of HUVECs because of a significant increase in Ang-1 secretion (Piard et al., 2019). Here, the small size (∼360 μm) of gelatin microspheres provided an appropriate distance. The paracrine effect of encapsulated rADSCs through exosomes was proved by both *in vitro* coculture (Figure 2F) and *in vivo* wound healing results (Figures 7C,D). Exosomes from MSCs were internalized by other cells and activated several signaling pathways, including Akt, ERK, and STAT3, enhancing proliferation and migration of fibroblasts and also angiogenesis (Shabbir et al., 2015). Furthermore, the 3D culture of ADSCs was found to improve the paracrine effect (Tomaszewski et al., 2019). Hence, for the rADSC/MS group, wound healing was significantly improved (Figure 4).

Recent studies found that *in vitro* co-encapsulation of ADSCs with early-stage follicles in an alginate-based 3D culture system supported follicular development through secretion of cytokines that promoted follicular survival, antrum formation, and meiotic competence (Green et al., 2019). Here, regeneration of hair follicles in the rADSC/MS group was much faster, whereas significantly more fluorescent cells around hair follicles than other groups were detected; these findings indicated that rADSCs might participate in the formation of follicles during wound healing. New hair follicles were reported to reprogram myofibroblasts that were abundant in dermal scar tissue to differentiate into adipocytes by activating the BMP-ZFP423 pathway during mouse wound healing. The depletion of myofibroblasts has been viewed as a main anti-scarring strategy (Plikus et al., 2017). Here, adipocytes were detected at the area close to the new hair follicles for both the MS and rADSC/MS groups on day 14 (Supplementary Figure S6), which might originate from myofibroblasts. Thus, it was reasonable that the deposition of well-organized collagen fibers was the fastest in the rADSC/MS group, as the regeneration of hair follicles was the earliest (Figure 4).

With a high specific surface area and appropriate *in vivo* degradation rate, gelatin microspheres facilitated tissue infiltration, enabling granulation tissue to grow tightly around these microspheres. Therefore, the presence of rADSCs in the gelatin matrix further contributed to the fastest wound closure, angiogenesis, collagen deposition, and peripheral nerve recovery. Hence, the rADSC/MS group showed better performance on improving diabetic wound healing than only gelatin microspheres.

## Conclusion

Gelatin microspheres were developed for local delivery of rADSCs to promote diabetic wound healing. Uniform, well-dispersed gelatin microspheres with desired biocompatibility and degradation properties were obtained, which provided a suitable microenvironment for rADSCs survival, avoiding direct exposure to the complex microenvironment of diabetic wounds. Because of MMP-mediated degradation, gelatin microspheres may improve the local microenvironment of diabetic wounds, leading to faster and better wound healing when compared with the blank group. The encapsulation of rADSCs in the rADSC/MS group promoted M2 polarization and peripheral nerve recovery and further facilitated angiogenesis and collagen and follicle formation, ultimately accelerating wound healing. The degradation rate of the rADSC/MS group matched well with tissue regeneration. Nevertheless, these data still could not provide direct evidence regarding whether rADSCs differentiated into adult somatic cells. Further investigations were needed. Taken together, gelatin microspheres showed great potential as a stem cell delivery vehicle and also as scaffold materials to improve diabetic wound healing.

## Data Availability

The original contributions presented in the study are included in the article/Supplementary Material; further inquiries can be directed to the corresponding author.
