# Supplementary material for: Adaptive Gelatin Microspheres Enhanced Stem Cell Delivery and Integration With Diabetic Wounds to Activate Skin Tissue Regeneration
Source: Front Bioeng Biotechnol. 2022 Apr 1;10:813805. doi: 10.3389/fbioe.2022.813805 (PMC9011108; doi:10.3389/fbioe.2022.813805)
Supplement: Supplementary file 1 [file DataSheet1.docx]

Supporting information for

**Adaptive gelatin microspheres enhanced stem cell** **delivery and integration with diabetic wounds** **to activate skin tissue regeneration**

Ming Shi ^1, 2, 3^, Yunfen Gao ^1, 2^, Lim Lee ^4^, Ting Song ^1, 2^, Jianhua Zhou ^1,^ ^2^, Ling Yan ^4^, Yan Li ^1, 2,^ *

1. Guangdong Provincial Key Laboratory of Sensor Technology and Biomedical Instrument, School of Biomedical Engineering, Shenzhen Campus of Sun Yat-sen University, Shenzhen 518107, Guangdong, P.R. China

2. Guangdong Provincial Engineering and Technology Center of Advanced and Portable Medical Devices, Sun Yat-sen University, Guangzhou 510006, Guangdong, P.R. China

3. Department of Scientific Research Center, The Seventh Affiliated Hospital, Sun Yat-Sen University, Shenzhen 518107, P.R. China

4. Department of Plastic and Cosmetic Surgery, The Third Affiliated Hospital, Sun Yat-sen University, Guangzhou 510630, Guangdong, P. R. China

^*^Corresponding author: Yan Li, Telephone: +86-20-39332146, Fax: +86-20-39332146, Email: liyan99@mail.sysu.edu.cn


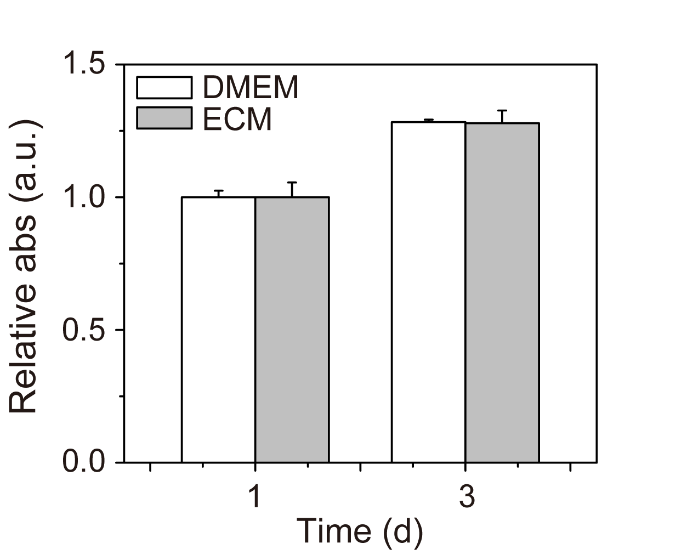


Figure S1. Monitoring proliferation of rADSCs on TCP and cultured in DMEM (routine medium for rADSCs) or ECM (routine medium for HUVECs) using CCK-8 assay; data = mean ± SD, n = 3.


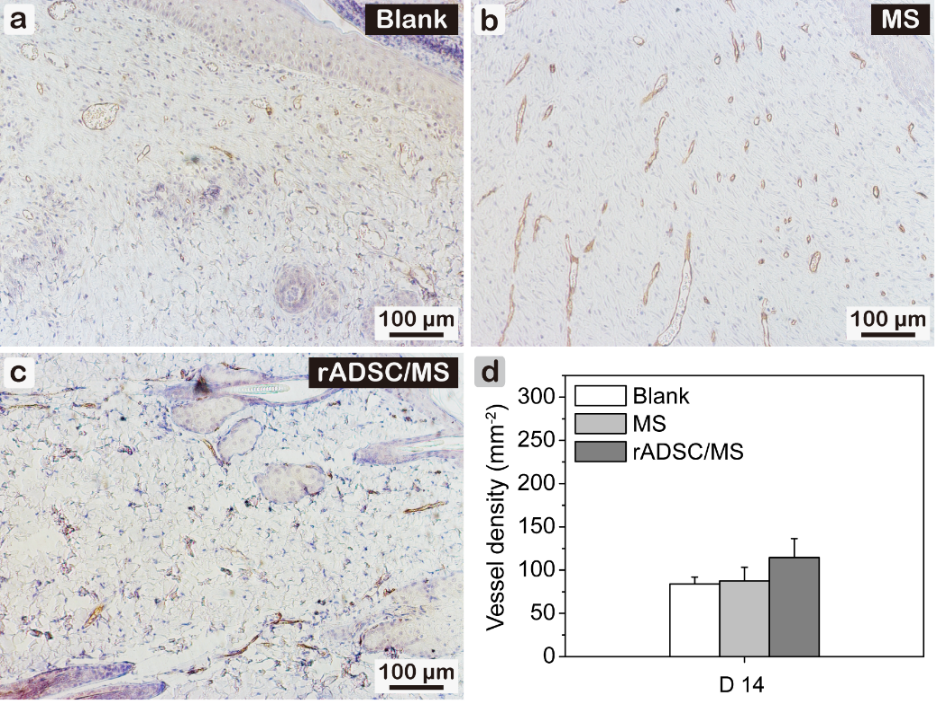


Figure S2. Representative light microscope images of CD31 immunohistochemical staining of wound sections on day 14 for (a) blank, (b) MS, (c) rADSC/MS groups, and (d) quantitative results of vessel density.


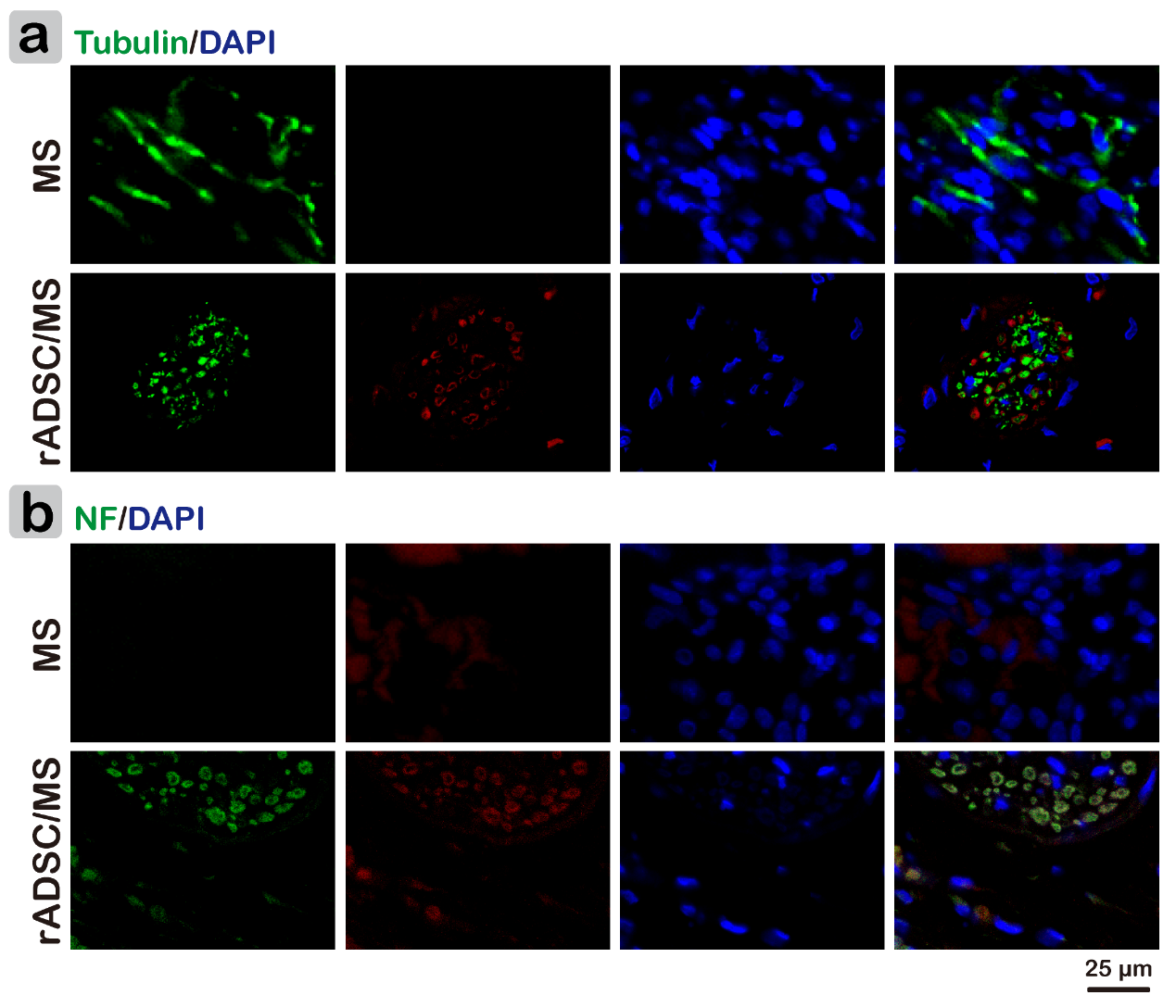


Figure S3. Immunofluorescence images of sections revealing the distribution of regenerated neurons (a) Tubulin and (b) axon (NF) in MS and rADSC/MS groups on day 14.


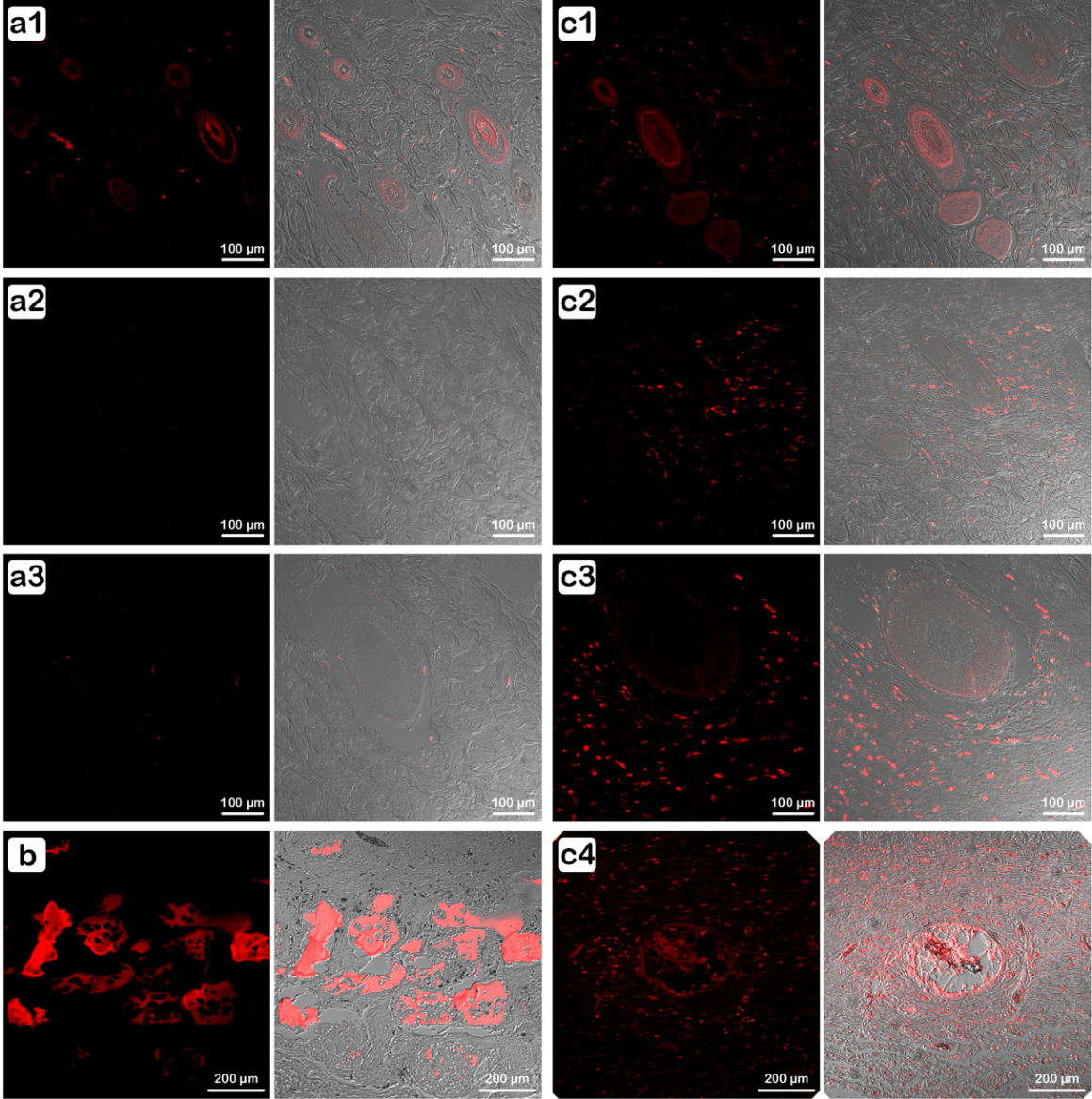


Figure S4. Representative CLSM images and merged with brightfield images at the same area of wound sections for (a) blank, (b) MS and (c) rADSC/MS groups on day 14. 1-3 were locations near the epidermis, middle layer of dermis and subcutaneous layer, respectively. c4 was the tissue surrounding microspheres in rADSC/MS group.


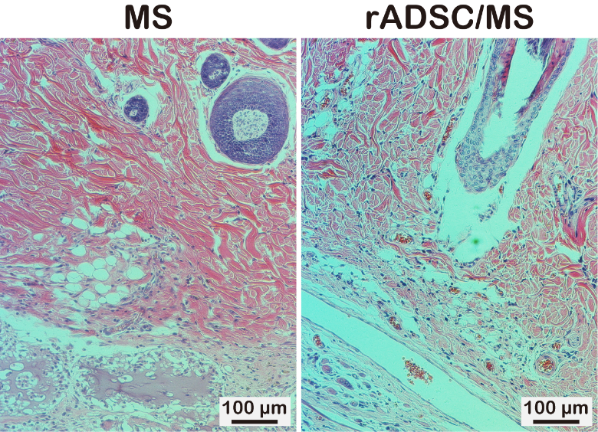


Figure S5. Adipocytes were detected at areas close to the new hair follicles for both MS and rADSC/MS group on day 14.


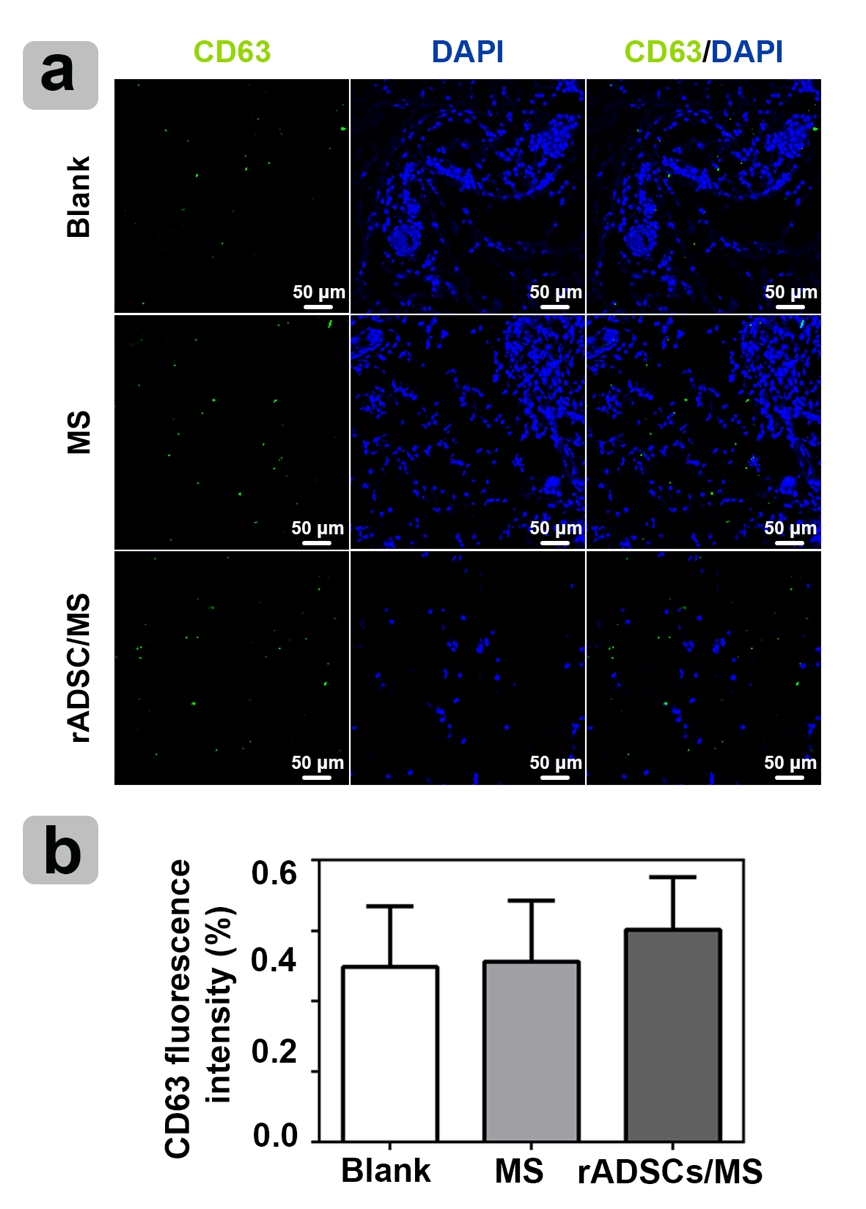


Figure S6. CD 63 fluorescence intensity of three groups on day 3. Data = mean ± SD, n = 3.
